# Using Immunization Data Systems to Track Zero-Dose Children in Bangladesh: Mixed Methods Exploratory Study

**DOI:** 10.2196/88951

**Published:** 2026-09-29

**Authors:** Samiha Yunus, Prem Singh, Alan Noble John, Elizabeth Oliveras, Md Jasim Uddin, Nurul Alam, Md Wazed Ali, Hemel Das, Chris Morgan

**Affiliations:** 1 Jhpiego Dhaka Bangladesh; 2 Jhpiego New Delhi India; 3 Jhpiego Maryland, MD United States; 4 Health Systems and Population Studies Division, icddr,b Dhaka Bangladesh

**Keywords:** immunization, zero-dose, vaccine, data system, children, Bangladesh

## Abstract

**Background:**

Zero-dose (ZD) children—those who receive no routine vaccines—remain highly vulnerable, with global evidence showing that they are concentrated in marginalized and hard-to-reach settings. In Bangladesh, stagnant full-immunization coverage, population mobility, and inconsistencies in administrative data complicate accurate estimation of ZD and underimmunized children. Gaps in the completeness, timeliness, and accuracy of routine Expanded Programme on Immunization (EPI) information systems further limit the ability to identify and target these populations.

**Objective:**

This study aimed to assess the completeness, timeliness, and accuracy of Bangladesh’s immunization data systems and to identify challenges and strategies for integrating ZD analysis into routine EPI information systems.

**Methods:**

We used a mixed methods exploratory descriptive qualitative approach comprising a desk review and stakeholder interviews conducted from February 2024 to April 2024. The desk review assessed program documents, gray literature, and published studies to map routine immunization data sources and their relevance for ZD analysis. Thirteen purposively selected stakeholders, including EPI and District Health Information Software 2 (DHIS2) managers, were interviewed using semistructured interview guides to gather insights on data availability, reporting practices, and system challenges. Information from both steps was synthesized using a structured checklist to evaluate completeness, timeliness, accuracy, and feasibility of ZD tracking across data sources. Data were analyzed manually using a deductive thematic approach.

**Results:**

Ten routine immunization data sources were identified, with DHIS2, the Rapid Convenience Monitoring (RCM) tool, and the EPI Coverage Evaluation Survey (CES) emerging as the primary systems for ZD tracking. Although most sources captured child-level data, the use of unique identifiers and household-level variables was inconsistent. Data quality and timeliness varied: DHIS2 faced denominator inaccuracies, CES and Bangladesh Demographic and Health Survey (BDHS) provided high-quality survey estimates, and RCM delivered real-time data from hard-to-reach areas. Multiple systems supported ZD identification, although each had limitations related to data completeness, sampling, geographic granularity, or operational constraints.

**Conclusions:**

Immunization data systems in Bangladesh are fragmented, with limited ZD tracking and predominantly district-level reporting. DHIS2, RCM, and Health and Demographic Surveillance System (HDSS) are the most promising platforms for routine ZD monitoring, offering nationwide integration, real-time field data, and detailed local analysis. Harmonizing these systems and combining data from multiple sources can improve targeted interventions, subnational monitoring, and equitable vaccine coverage.

## Introduction

Although immunization is one of the most effective and cost-efficient public health interventions, approximately 15 million children worldwide remained unvaccinated in 2023 [1]. These children, classified as zero-dose (ZD) children, received none of the routine childhood vaccines, leaving them highly vulnerable to preventable and potentially life-threatening diseases [1]. Systemic inequalities in immunization systems contribute to the lack of access to necessary vaccinations [2]. In low- and middle-income countries (LMICs), children who miss their first dose of the diphtheria, tetanus, and pertussis (DTP1) vaccination account for nearly half of all vaccine-preventable deaths [3]. Evidence reported that ZD children are usually concentrated in urban poor, remote rural, or conflict-affected areas, with more than 45% of the 9.7 million children residing in such geographic settings [4-6]. Evidence from a Country Learning Hub (CLH) rapid assessment report corroborates global trends to some extent in the context of Bangladesh [7]. The report highlights the heightened vulnerability of migrant and internally displaced populations, alongside specific demand-side barriers. It also points to common structural determinants observed globally, including low socioeconomic status, forced migration, homelessness, and religious or cultural marginalization [7].

In Bangladesh, coverage measured in terms of fully vaccinated children has fluctuated between 80% and 84% over the past decade, resulting in a persistently undervaccinated population of 16% to 20% [8]. Bangladesh’s Expanded Programme on Immunization (EPI) must reach underprivileged groups, as unvaccinated people are still susceptible to diseases such as measles and diphtheria [9]. Achieving high vaccination coverage and precisely determining the ZD burden are made more difficult by the nation’s diverse, hard-to-reach communities and urban migration [7,9]. Official estimates of DTP1, collated by the World Health Organization (WHO) and United Nations Children’s Fund (UNICEF), have fluctuated between 93% and 100% over the past decade, with administrative estimates often exceeding 100% [10]. Overestimated numerators or denominators can lead to errors in ZD or underimmunized estimates. Therefore, a more accurate estimation method is provided by the rapid assessment report, which also emphasizes the necessity of resolving issues with health information systems to prioritize programs and monitor progress in ZD reduction [7].

Routine EPI information systems are foundational to immunization programs, supporting the planning, implementation, and evaluation of vaccination services. These systems routinely collect data from community and facility levels and play a critical role in identifying areas with low coverage, including ZD children, to guide targeted and equitable interventions. In Bangladesh, however, gaps in data completeness, timeliness, and accuracy may hinder the ability to effectively identify and reach ZD children. This study thus aimed to conduct a comprehensive review of Bangladesh’s immunization data landscape to assess the completeness, timeliness, and accuracy of existing data sources; evaluate their strengths and limitations; and identify feasible strategies for integrating ZD analysis into routine EPI information systems.

## Methods

### Study Design

We adopted a mixed methods exploratory descriptive qualitative approach to assess the routine immunization data landscape, with a focus on ZD children in Bangladesh. Data collection was conducted using multiple methods, including a desk review and interviews conducted between February 2024 and April 2024. First, a rapid desk review was undertaken to identify existing immunization-related data sources and relevant stakeholders. Furthermore, we conducted interviews with stakeholders as part of the qualitative component to gather in-depth insights.

### Desk Review

We conducted an in-depth desk review of program documents, gray literature, and published empirical studies related to immunization data sources in Bangladesh. The primary sources used to identify these data sources were official websites, reports, and publications. To identify the appropriate data sources, we followed multiple processes. Using keywords such as immunization, immunization data, ZD children, EPI, vaccination, coverage, hard-to-reach areas, and Bangladesh, we searched for relevant documents and websites using Google. We identified relevant websites, dashboards, literature, and reports and thoroughly reviewed each document and the respective organizations’ websites. The review focused on gathering information about the characteristics of each data source, availability of data on ZD children, reporting frequency, and the strengths and limitations of each source.

### Qualitative Interviews

We conducted interviews with key stakeholders involved in immunization systems and in strengthening these systems in Bangladesh. The interviews complemented the desk review. Participants were selected purposively based on their relevance to the identified immunization systems in Bangladesh. Initially, we contacted potential participants and shared the study objectives. Subsequently, interviews were conducted at times and locations convenient for the participants. For example, we interviewed EPI managers responsible for the District Health Information Software 2 (DHIS2; HISP Centre), as they are tasked with routinely updating the immunization data, including data on ZD children. The aim of these interviews was to gain valuable insights into the data sources used by vaccination programs, with a particular focus on information related to ZD children. Moreover, we obtained the necessary credentials to access the identified websites, as they were not publicly available and required proper authorization for access. A total of 13 interviews were conducted using a semistructured interview guide.

### Review of Data Sources

We combined the insights gained from the interviews and the desk review to assess the selected websites and documents. Using the developed checklist, each data source was analyzed using specific themes to evaluate its capacity for ZD analysis at the national, administrative division, and district levels. We assessed the feasibility of ZD tracking, the availability of data on ZD children, and reporting frequency and compared the sources based on these features. Furthermore, we explored the strengths and limitations associated with each data source.

### Data Analysis

We conducted a descriptive and deductive thematic analysis. After the completion of the interviews, audio recordings and interview notes were promptly translated into English. Thematic analysis was conducted using the 6-phase approach developed by Braun and Clarke [11]. Using this approach, responses were categorized under key themes, including the availability of data on ZD children, data quality, timeliness, strengths, and limitations. Coding was conducted independently by 2 study team members, with regular meetings held to compare, discuss, and reconcile differences in coding. Discrepancies were resolved through discussion and consensus, with oversight from other team members. To enhance rigor, peer debriefing was conducted throughout the analysis to support interpretation and ensure consistency in theme development. Triangulation was achieved by comparing findings across the desk review and interviews. The data were analyzed manually and presented in tabular format to facilitate comparison and interpretation.

### Ethical Considerations

This study was part of the CLH activities, and ethics approval for the study was obtained from the institutional review board of the International Centre for Diarrhoeal Disease Research, Bangladesh (icddr,b) before data collection (PR#22170). Informed written consent was obtained from all participants prior to the interviews, and the confidentiality and anonymity of the information they provided were ensured.

## Results

### Sources of Routine Immunization Data and Relevant Stakeholders

We identified a total of 10 immunization data sources in Bangladesh [12-23]. Among them, 5 (50%) data sources were online-based systems used primarily for routine data collection and monitoring. A total of 4 (40%) data sources were document-based, published periodically as survey or report outputs. The remaining 1 (10%) data source involved the use of the Rapid Convenience Monitoring (RCM) tool, a digital tool for household monitoring and immunization session assessment. The 3 major sources of routine immunization data in Bangladesh are DHIS2, the RCM tool, and the EPI Coverage Evaluation Survey (CES). DHIS2 is a nationally scaled, open-source digital health management information system covering all public health facilities. RCM, used through the WHO–Surveillance and Immunization Medical Officer (SIMO) network, provides real-time data from hard-to-reach and underserved areas via digital tools. CES is a periodic survey using multistage cluster sampling to evaluate immunization coverage and service quality through household interviews and record reviews. The list of identified data sources for routine immunization in Bangladesh is provided in Table 1.

**Table 1:** Sources of routine immunization data and relevant stakeholders.

Data sources	Data source’s availability	Stakeholders consulted
District Health Information Software 2	Website	EPI^a^ managers
EPI Coverage Evaluation Survey 2019	Report	WHO^b^
Routine immunization monitoring and supervision data	Report	WHO
Bangladesh Demographic and Health Survey	Demographic and Health Surveys website	EPI managers and partners
Health and Demographic Surveillance System data	Report—MATLAB	International Centre for Diarrhoeal Disease Research, Bangladesh
Effective Vaccine Management	Report	WHO and United Nations Children’s Fund
Vaccine-preventable disease surveillance data	Report	Government officials
Adverse events following immunization surveillance data	Website	Directorate General of Drug Administration, Bangladesh
Digital tools for household monitoring and immunization session assessment	Tools supplied by the Ministry of Health and Family Welfare	EPI managers
Geospatial information systems overviews	Website	WHO; Bangladesh Geographic Information System Portal

^a^EPI: Expanded Programme on Immunization.

^b^WHO: World Health Organization.

### Key Features of Data Sources

Most systems, including DHIS2, CES, RCM, Bangladesh Demographic and Health Survey (BDHS), and Health and Demographic Surveillance System (HDSS), collect detailed child-level information, including child identifiers, age, sex, and health card or vaccination registry details. However, unique identifiers are inconsistently used across systems; only DHIS2, BDHS, and HDSS incorporate them. Birth order is recorded solely by BDHS. BDHS is the most comprehensive source for capturing household-level variables such as guardian’s education and wealth quintile. CES and RCM also partially collect these variables, while DHIS2 and HDSS have limited coverage. CES includes questions about vaccination side effects, the number of required visits, and the source of vaccination. BDHS covers general awareness about immunization, but other systems have limited or no data on these indicators. CES uniquely captures information about hard-to-reach areas, while BDHS includes geographic coordinates of health facilities, which can support spatial mapping and planning. Only the vaccine-preventable disease (VPD) surveillance system records comprehensive operational elements such as training, logistic support, storage and cold chain capacity, adverse event following immunization (AEFI), population coverage, and referral systems. These elements are not addressed by the other systems. More specific information is available in Table 2.

**Table 2:** Key features of different data sources.

Data elements		District Health Information Software 2	Coverage Evaluation Survey 2019	Rapid Convenience Monitoring	Bangladesh Demographic and Health Survey 2017-2018	Health and Demographic Surveillance System	Vaccine-preventable disease surveillance
**Child-level data**							
	Unique identifier	✓		✓	✓		
	Child identifier	✓	✓	✓	✓	✓	✓
	Card, registry, or history	✓	✓	✓	✓		✓
	Age	✓	✓	✓	✓	✓	
	Sex	✓	✓	✓	✓	✓	
	Birth order				✓		
	Anthropometric data	✓		✓	✓		
	Guardian’s education		✓		✓	✓	
	Wealth quintile		✓		✓	✓	
**Knowledge about vaccination**							
	Side effect of vaccination		✓				
	Knowledge of number of visits		✓				
	Source of vaccination		✓				
	Vaccination awareness^a^					✓	
**Health facility information**							
	Hard-to-reach area^b^		✓				
	Geocoordinates of health facilities					✓	
**Immunization services**							
	Training						✓
	Logistic support						✓
	Storage capacity						✓
	Cold storage						✓
	Population coverage						✓
	Adverse event following immunization						✓
	Referral system						✓

^a^Vaccination awareness includes which vaccine is being received, knowledge about pentavalent vaccination, how the participant learned about pentavalent vaccination, and knowledge about the next vaccination.

^b^Hard-to-reach areas are geographically difficult-to-access areas where the delivery of routine immunization services is constrained by factors such as remote locations, poor transportation, riverine (*char*) settings, and hill tract areas with limited access to health facilities.

### Data Quality, Integrity, Timeliness, and ZD Tracking

The DHIS2 system faces restrictions due to denominator inaccuracies, as it relies on projected census data that may not accurately reflect actual facility catchment populations. In contrast, the EPI-CES and the BDHS are considered high-quality sources, benefiting from rigorous sampling methods and experienced survey teams. Routine immunization monitoring provides useful insights, but the effectiveness of the data depends heavily on adequate sample sizes for sessions and households, especially when targeting ZD populations. The HDSS contributes high-quality data through regularly shared summaries and accessible datasets, supporting robust analysis for both researchers and policymakers.

DHIS2 reports EPI data on a monthly or quarterly basis, depending on the health system level being monitored. The EPI-CES has a reporting frequency of 1 to 4 years, which allows in-depth analysis of national immunization coverage and access. RCM provides real-time data through tools such as Power BI (Microsoft Corp) and KOBO (Kobo Organization), enabling immediate access for EPI managers to track vaccination sessions and household monitoring. The BDHS releases data every 3 to 5 years, providing a valuable tool for long-term monitoring of immunization progress.

Several data sources contribute to identifying and understanding ZD children in Bangladesh. DHIS2 tracks key vaccinations such as Penta1, serving as a proxy for ZD identification, although limitations in data quality may affect accuracy. The EPI-CES provides detailed, disaggregated data on immunization coverage, enabling robust ZD estimates. The RCM tool identifies ZD and undervaccinated children while capturing community-level barriers to vaccine uptake. The BDHS offers nationally representative data to estimate ZD prevalence among children aged 12 to 23 months. Finally, the HDSS provides granular, area-specific data on immunization status and timeliness. Together, these sources offer a comprehensive understanding of ZD children, supporting targeted strategies to improve vaccine coverage. More specific information is available in Table 3.

**Table 3:** Data quality, integrity, timeliness, and zero-dose (ZD) tracking.

Data sources	Data quality	Timeliness	ZD tracking capacity	Data entry access level
District Health Information Software 2	Moderate; completeness concerns, especially for dropout tracking	Real-time data entry but often delayed reporting from facilities	Partial; potential with unique IDs but limited implementation	Facility-level data entry by health workers
Expanded Programme on Immunization Coverage Evaluation Survey	High; based on rigorous methodology and a large sample	Retrospective; typically conducted every 5 years	Yes; ZD children can be identifiable through survey questions	Trained survey teams collect and enter data through centralized survey management
Rapid Convenience Monitoring	Moderate; relies on field monitoring, with some inconsistency	Monthly to quarterly monitoring cycles	No direct tracking; purposive sampling of ZD clusters is needed	Field supervisors or the Surveillance and Immunization Medical Officer network enter data using digital tools
Bangladesh Demographic and Health Survey	High; standardized survey protocol with strong sampling	Every 3 to 5 years	Yes; allows identification based on receipt of the first dose of the diphtheria, tetanus, and pertussis vaccine	Dedicated national survey teams collect and enter data
Health and Demographic Surveillance System	High; maintained by the International Centre for Diarrhoeal Disease Research, Bangladesh, using strict protocols	Continuous, although not nationally representative	Yes; detailed child histories enable ZD estimation	Site-based, trained field staff enter longitudinal household data
Vaccine-preventable disease surveillance	Moderate; captures operational data but is not designed for detailed child tracking	Periodic; not real time	No	Facility-based reporting by clinicians and surveillance officers

### Implementation-Related Strengths and Challenges

The integration of DHIS2 across Bangladesh’s health system enabled monthly and quarterly tracking of immunization coverage, particularly with respect to ZD data, down to local levels, including Upazila and City Corporation facilities, thus supporting informed decision-making. The CES 2019 provided detailed data on immunization coverage through rigorous sampling, addressing denominators and incorporating factors such as vaccinated and unvaccinated children. In terms of real-time supervision, RCM facilitated the prompt detection of ZD children, focusing on potential clusters and capturing reasons for nonvaccination. Additionally, the BDHS 2017-2018 offered comprehensive, population-representative data that were useful for hypothesis generation, while HDSS captured unique factors, such as migration. Moreover, VPD surveillance systems and regulatory frameworks strengthened vaccine delivery and safety monitoring.

The constraints of various systems regarding immunization data tracking and ZD identification were notable (Table 3). DHIS2 faced challenges such as potential reporting bias, restricted multilevel data, reliance on aggregate data with limited individual-level insights, and the absence of household-level ZD tracking. The CES 2019 had several limitations, including its inability to provide subdistrict estimates, reliance on small clusters, high costs, recall bias, and a lack of sociobehavioral insights. Similarly, RCM depended on external resources, was at risk of biased estimates due to convenience sampling, and was restricted to district-level data. The BDHS 2017-2018 relied on immunization cards and lacked data on nonvaccination. HDSS was limited by aggregated data and restricted public access. Finally, VPD systems faced workforce shortages, geographic inequities, and cold chain gaps. More specific information is available in Table 4.

**Table 4:** Implementation-related challenges and scalability of health and immunization monitoring systems.

Data sources	Health workforce	Infrastructure	Data capture quality	Timeliness	Monitoring and supervision	Scalability
District Health Information Software 2	High workload on health workers; limited training	Poor infrastructure in some areas	Risk of reporting bias; limited individual-level data	Routine reporting (monthly or quarterly); delays in facility reporting	Limited data-use feedback loops	High; nationally implemented but needs quality strengthening
Coverage Evaluation Survey 2019	Requires trained survey teams	Strong logistical requirements	High-quality but subject to recall bias	Periodic (every 1 to 5 years); not real time	Strong centralized supervision during the survey	Low; resource intensive and costly
Rapid Convenience Monitoring	Dependence on field staff capacity	Requires digital tools and connectivity	Inconsistent reporting; potential sampling bias	Monthly to quarterly cycles	Strong reliance on supervisory effectiveness	Moderate; depends on resources and supervision
Bangladesh Demographic and Health Survey	Requires experienced national survey teams	High survey logistics requirements	High quality but subject to recall bias	Every 3 to 5 years; long intervals	Strong national survey oversight	Low; not designed for routine monitoring
Health and Demographic Surveillance System	Requires skilled field surveillance staff	Site-based infrastructure only	High-quality longitudinal data; limited generalizability	Continuous data collection	Strong site-level monitoring systems	Low to moderate; expansion is resource intensive
Vaccine-preventable disease surveillance	Clinical and surveillance staff burden	Health facility–based systems; cold chain dependency	Incomplete community capture; operational data focus	Periodic; not real time	Variable supervision across facilities	Moderate; complements other systems

## Discussion

### Principal Findings and Comparison With Prior Work

This study found that Bangladesh has multiple immunization data systems with complementary strengths, including routine digital reporting systems, periodic surveys, and surveillance platforms. However, no single system provides complete, timely, and consistently high-quality data for identifying and tracking ZD children. Although systems such as DHIS2, CES, BDHS, HDSS, and RCM each contribute valuable information, gaps remain in data completeness, standardization, and individual-level tracking. Timeliness also varies widely across systems, ranging from real-time reporting to survey cycles spanning multiple years. Overall, the findings highlight the need for better integration and harmonization of existing data sources to support effective ZD monitoring within routine EPI systems.

Among the identified sources, 1 major data source, the EPI-CES, primarily focuses on routine immunization services delivered through fixed-site facilities. In contrast, another significant source, the EPI Outreach Monitoring System, is concerned with immunization coverage achieved through outreach activities, particularly among hard-to-reach and underserved populations. The lack of an integrated and comprehensive national data system that simultaneously captures both facility-based and outreach-based immunization services poses a challenge for monitoring equitable coverage and planning targeted interventions [24]. This fragmentation in immunization data systems has also been noted in previous assessments, which emphasized the need for harmonized and timely data collection tools to improve vaccine coverage monitoring and program planning in LMICs, including Bangladesh [25,26].

Furthermore, we observed that multiple health and vaccination monitoring systems in Bangladesh use different approaches to data collection and immunization coverage tracking. Each system contributes uniquely to identifying ZD children and monitoring vaccination service delivery. This diversity highlights both the strengths and challenges of having multiple, often uncoordinated, data sources for immunization tracking. The disintegration of these systems may impede comprehensive coverage analysis and strategic planning; however, when effectively harmonized, they also offer valuable opportunities for designing and implementing more targeted and context-specific interventions [27,28].

The study also revealed that DHIS2 has played a crucial role in monitoring vaccination coverage at different levels of the health system, such as in Upazila Health Complexes. DHIS2 is well suited for capturing and managing health data, including ZD data. As part of Bangladesh’s health information infrastructure, DHIS2 supports routine tracking of immunization data, enabling timely, data-driven decision-making. The integration of features into DHIS2 to capture ZD data has accelerated its potential. Like Bangladesh, several countries, including Mozambique, Nigeria, and the Democratic Republic of the Congo, have integrated features into their DHIS2 platforms to identify and monitor ZD [29,30]. Globally, DHIS2 is widely adopted in more than 70 countries to strengthen health information systems and improve public health program management, including immunization services [31].

On the other hand, although CES 2019 provides comprehensive immunization data at the household level, its reliance on surveys and retrospective data collection introduces potential biases and scalability challenges. Recall bias and the inability to provide real-time data limit the accuracy and timeliness of the information. Additionally, the resource-intensive nature of the survey makes it difficult to scale or repeat on a routine basis. Thus, CES is valuable but needs to be paired with real-time systems for more effective monitoring and interventions [13].

In addition, RCM is identified as a useful tool for filling immunization gaps as they appear because it allows real-time tracking of ZD children and the causes of nonvaccination. However, it has limited scope in particular regions, which reduces its generalizability at the national level [32]. In contrast, population-representative data from the BDHS 2017-2018 makes regional and temporal comparisons easier [33]. However, its reliance on immunization records, combined with the lack of data on nonvaccination, highlights a significant knowledge gap in understanding the causes of missed vaccinations. Although its methodology provides valuable insights, updates to the immunization tracking strategy are essential to address contemporary vaccination challenges. Understanding the in-depth reasons behind the high proportion of ZD children is crucial for effective policy formulation [34].

We found that HDSS provides valuable insights into population dynamics such as migration and their impact on vaccination coverage. However, its aggregated data limit its use for identifying ZD children at the household level. Integrating HDSS with more granular sources such as CES, BDHS, or RCM could improve the precision of immunization strategies. Enhancing HDSS to capture disaggregated data may further support efforts to reach underserved populations and strengthen vaccine equity. Moreover, although strong regulatory frameworks for VPD surveillance systems enhance vaccination distribution and safety monitoring, persistent systemic issues, such as cold chain limitations and geographic disparities, remain major barriers to achieving equitable coverage [35].

To strengthen monitoring systems, it will be crucial to integrate national platforms such as DHIS2 and BDHS with the localized insights offered by CES 2019 and RCM [13,15]. Although DHIS2 and BDHS capture broader immunization trends, CES and RCM provide critical microlevel data that can support the identification and understanding of ZD children [7]. However, achieving equitable vaccine coverage also demands overcoming systemic barriers such as data inaccessibility, workforce shortages, and geographic disparities [36,37]. Incorporating sociobehavioral insights into immunization tracking systems can further enhance their responsiveness to community-level barriers. A harmonized, multisource approach holds significant potential for reaching ZD populations, particularly in underserved and hard-to-reach areas [33].

### Limitations

This study has several limitations. First, it did not follow a systematic review methodology such as PRISMA (Preferred Reporting Items for Systematic Reviews and Meta-Analyses), which may affect the comprehensiveness and reproducibility of the evidence synthesis process. Second, the qualitative analysis did not include direct quotations or attributed participant perspectives, which may have limited the depth of interpretation of stakeholder views. Finally, restricted access to certain immunization data sources limited the breadth of the analysis.

### Strengths

Despite these limitations, this study provides a comprehensive assessment of Bangladesh’s immunization data ecosystem by systematically mapping multiple routine, survey-based, and surveillance data systems. It combines a documentary review with stakeholder consultations, enabling triangulation of findings. The inclusion of diverse data sources allows a holistic evaluation of data quality, timeliness, and utility for ZD tracking and programmatic decision-making.

This study highlights clear opportunities to strengthen Bangladesh’s immunization data ecosystem through improved integration and harmonization of existing systems. Linking routine platforms such as DHIS2 with survey and surveillance data could enhance data completeness, validation, and triangulation for ZD tracking. Expanding the use of unique identifiers and improving interoperability across systems would support better individual-level tracking. Incorporating contextual and sociobehavioral data into routine systems could further improve the targeting of underserved populations and inform more responsive immunization strategies.

### Conclusions

This study highlights fragmentation across immunization data systems in Bangladesh, where multiple platforms capture different aspects of immunization, but none provide a complete, timely, and disaggregated picture of ZD children. Limitations include weak system integration, variable data quality (particularly denominator accuracy), and limited availability of subdistrict-level data, which constrain routine monitoring and equity-focused decision-making. DHIS2 shows the strongest potential for routine ZD tracking, while RCM offers more granular, context-specific insights and HDSS supports in-depth subpopulation analysis; CES provides periodic validation but requires better disaggregation and integration with routine systems. Strengthening interoperability across DHIS2, RCM, and CES; standardizing core indicators; improving data quality assurance; and enabling routine use of subdistrict-level data are essential steps to support a harmonized, multisource approach for improving ZD identification and immunization equity in Bangladesh.

## Data Availability

The datasets used and/or analyzed during the current study are available from the corresponding author upon reasonable request.

## Abbreviations

AEFI: adverse event following immunization
BDHS: Bangladesh Demographic and Health Survey
CES: Coverage Evaluation Survey
CLH: Country Learning Hub
DHIS2: District Health Information Software 2
DTP1: first dose of the diphtheria, tetanus, and pertussis
EPI: Expanded Programme on Immunization
HDSS: Health and Demographic Surveillance System
icddr,b: International Centre for Diarrhoeal Disease Research, Bangladesh
LMIC: low- and middle-income country
PRISMA: Preferred Reporting Items for Systematic Reviews and Meta-Analyses
RCM: Rapid Convenience Monitoring
SIMO: Surveillance and Immunization Medical Officer
UNICEF: United Nations Children’s Fund
VPD: vaccine-preventable disease
WHO: World Health Organization
ZD: zero dose
